# *Escherichia coli* Braun Lipoprotein (BLP) exhibits endotoxemia – like pathology in Swiss albino mice

**DOI:** 10.1038/srep34666

**Published:** 2016-10-04

**Authors:** Chikkamenahalli Lakshminarayana Lakshmikanth, Shancy Petsel Jacob, Avinash Kundadka Kudva, Calivarathan Latchoumycandane, Puttaraju Srikanta Murthy Yashaswini, Mosale Seetharam Sumanth, Cassiano F. Goncalves-de-Albuquerque, Adriana R. Silva, Sridevi Annapurna Singh, Hugo C. Castro-Faria-Neto, Sandeep Kumble Prabhu, Thomas M. McIntyre, Gopal Kedihithlu Marathe

**Affiliations:** 1 Department of Studies in Biochemistry, University of Mysore, Manasagangothri, Mysore – 570 006, Karnataka, India; 2 Department of Veterinary and Biomedical Sciences, Center for Molecular Immunology and Infectious Disease and Center for Molecular Toxicology and Carcinogenesis, 115 Henning Building, The Pennsylvania State University, University Park, PA 16802, USA; 3 Department of Cellular and Molecular Medicine, Cleveland Clinic Lerner Research Institute, 9500 Euclid Avenue, Cleveland, Ohio 44195, USA; 4 Department of Protein Chemistry & Technology, Central Food Technological Research Institute/CSIR, Mysore – 570 020, Karnataka, India; 5 Laboratótio de Imunofarmacologia, Instituto Oswaldo Cruz, Fundação Oswaldo Cruz, Rio de Janeiro, RJ, 21045-900, Brazil

## Abstract

The endotoxin lipopolysaccharide (LPS) promotes sepsis, but bacterial peptides also promote inflammation leading to sepsis. We found, intraperitoneal administration of live or heat inactivated *E. coli* JE5505 lacking the abundant outer membrane protein, Braun lipoprotein (BLP), was less toxic than *E. coli* DH5α possessing BLP in Swiss albino mice. Injection of BLP free of LPS purified from *E. coli* DH5α induced massive infiltration of leukocytes in lungs and liver. BLP activated human polymorphonuclear cells (PMNs) *ex vivo* to adhere to denatured collagen in serum and polymyxin B independent fashion, a property distinct from LPS. Both LPS and BLP stimulated the synthesis of platelet activating factor (PAF), a potent lipid mediator, in human PMNs. In mouse macrophage cell line, RAW264.7, while both BLP and LPS similarly upregulated TNF-α and IL-1β mRNA; BLP was more potent in inducing cyclooxygenase-2 (COX-2) mRNA and protein expression. Peritoneal macrophages from TLR2^−/−^ mice significantly reduced the production of TNF-α in response to BLP in contrast to macrophages from wild type mice. We conclude, BLP acting through TLR2, is a potent inducer of inflammation with a response profile both common and distinct from LPS. Hence, BLP mediated pathway may also be considered as an effective target against sepsis.

Sepsis is a life-threatening organ dysfunction caused by a dysregulated host response to infection^1^. It annually accounts for 19 million cases worldwide^2^ and 1,400 deaths per day^3^. Sepsis, therefore causes more deaths than breast cancer, prostate cancer and HIV/AIDS combined^4^. To date, more than hundred clinical trials conducted against sepsis have failed and there is no single drug to treat sepsis^5^. One possibility behind the disappointing clinical trials may be the failure to target critical mediator(s) in this complex syndrome^5^,^6^.

Systemic inflammation is the hallmark of sepsis^7^ and in majority of the cases intact bacteria can be implicated as the culprits^8^. However, negative blood cultures are often obtained from severe septic patients due to various reasons^9^. Therefore, the presence of a live bacterium is not a pre-requisite for sepsis^10^. Accordingly, bacterial endotoxins such as lipopolysaccharide (LPS) are widely used to mimic the pathobiology of sepsis^11^.

It is estimated that single Gram negative bacterial cell contains two million molecules of LPS^12^, covering approximately 75% of the outer membrane surface^13^. LPS exerts its action through Toll-like receptor 4 (TLR4)^14^, a pattern recognizing receptor that ligates structurally conserved pathogen associated molecular patterns (PAMPs) of microbial origin^15^. LPS additionally requires the association of LPS binding protein (LBP)^16^ and CD14 signaling components^17^ with TLR4 to initiate its signaling. Activation of TLR4 leads to mobilization of the transcription factor NF-κB to the nucleus^18^, thereby stimulating induction of an array of mediators^19^,^20^ that induce and amplify the inflammatory responses. Attempts to abolish the inflammatory insult using monoclonal antibodies against LPS^21^,^22^, its receptor^23^,^24^, and downstream signaling molecules^25^,^26^,^27^, have been unsuccessful. This suggests LPS may not be the sole endotoxin of Gram negative bacteria. This is also evident from a study by Freudenberg *et al*.^28^ where mouse strains with mutant tlr4 (C3H/HeJ and C57BL/10ScCr) are resistant to LPS action, but remain susceptible to infection by intact Gram-negative bacteria. Moreover, in the past, commercial LPS was often contaminated with lipoproteins that were also proinflammatory^29^. However, recently available LPS preparations are free of contaminats.

One abundant proinflammatory component of *E. coli* membranes is the Braun lipoprotein (BLP)^30^. Each *E. coli* contains 10^5^ molecules of BLP^31^, and so is the next most abundant structural component of the bacterial membrane. BLP is a ~7.2 kDa lipopeptide with a modified N-terminal cysteine residue bearing three palmitoyl residues making the structure a triacylcysteinyl-modified peptide^32^. Proteins bearing the modified N-terminal cysteine residues are known to be immunomodulatory and activate macrophages^33^, lymphocytes^34^ and endothelial cells^30^ as efficiently as LPS. In fact, there is a near complete concordance in the level of gene induction by LPS and BLP^30^. Unlike LPS, BLP acts via a related Toll-like receptor 2 (TLR2) and is independent of CD14 and LPS-binding protein requirement. BLP is more potent on a molar basis than its synthetic structural analogue Pam3CysSK4, a commercially available TLR2 agonist^30^, so other components of the protein contribute to TLR2 activation.

Although, the inflammatory roles of BLP have been shown using cultured endothelial cells^30^, its inflammatory roles in animal models have not been evaluated, though its structural analogue Pam3CysSK4 indicates the essential role of protein^35^,^36^. In this study, we have purified BLP free of LPS and assessed its inflammatory properties both *in vitro* and *in vivo*. We find purified BLP not to be inferior to LPS in both environments and in fact, it is more potent than LPS in eliciting an inflammatory response in some assays. Thus, BLP represents one of the unrecognized culprits in sepsis, and signaling events initiated by it may offer new avenues for therapeutic interventions.

## Results

### *E. coli* lacking BLP are less toxic than the parental strain with BLP in Swiss albino mice

**Figure 1:**
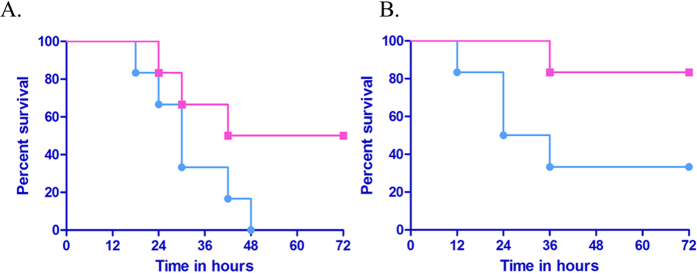
Lethality of Swiss albino mice in response to the administration of either live or heat inactivated *E. coli* DH5α (BLP +ve) and JE5505 (BLP −ve). 1 × 10^9^ CFU of either (**A**) live or (**B**) heat inactivated DH5α (Blue line) and JE5505 (pink line) strains *of E. coli* were injected intraperitoneally in a total volume of 500 μl in PBS to groups of Swiss albino mice (n = 6). Survival time was monitored for up to 72 hours. These results are representative of two independent experiments. p ≤ 0.08 between the groups as determined by log-rank test.

To understand the relative contribution of BLP in inducing lethality, we intraperitoneally injected 1 × 10^9^ CFU of live or heat inactivated *E. coli* that contains BLP (DH5α) or a mutant (JE5505) lacking this lipoprotein into Swiss albino mice and the survival time was monitored for 72 hours. We found, 100% of animals injected with 1 × 10^9^ CFU of live *E. coli* (DH5α) died within 48 hours while, 33.33% animals administered with 1 × 10^9^ CFU of heat inactivated *E. coli* (DH5α) survived at the end of the experiment (Fig. 1A). In contrast, 50% of animals injected with 1 × 10^9^ CFU of live *E. coli* that lack BLP (JE5505) survived at the end of the experiment while, only 16.66% mortality was observed in animals injected with heat inactivated *E. coli* (JE5505) (Fig. 1B). These results suggest that BLP may contribute to mortality from systemic *E. coli*.

In a similar experiment conducted to evaluate the effect of various concentrations of *E. coli* strains (1 × 10^6^ CFU to 1 × 10^12^ CFU) that were heat killed by autoclaving showed no lethality when 1 × 10^6^ CFU of either strain was injected intrapritoneally. However, 33.33% of animals injected with 1 × 10^9^ CFU and 100% of animals injected with 1 × 10^12^ CFU of *E. coli* DH5α died within 24 hours while, no mortality was observed in animals injected with heat killed *E. coli* JE5505 even at a concentration of 1 × 10^12^ CFU at the end of the experiment (Data not shown).

### Purification and biophysical characterization of BLP from *E. coli* DH5α

BLP from *E. coli* DH5α was purified as described by Inouye *et al*.^37^ and Nielsen *et al*.^30^. For detailed information see Supplementary information.

### BLP activates isolated human PMNs in a serum and polymyxin B - independent fashion

**Figure 2:**
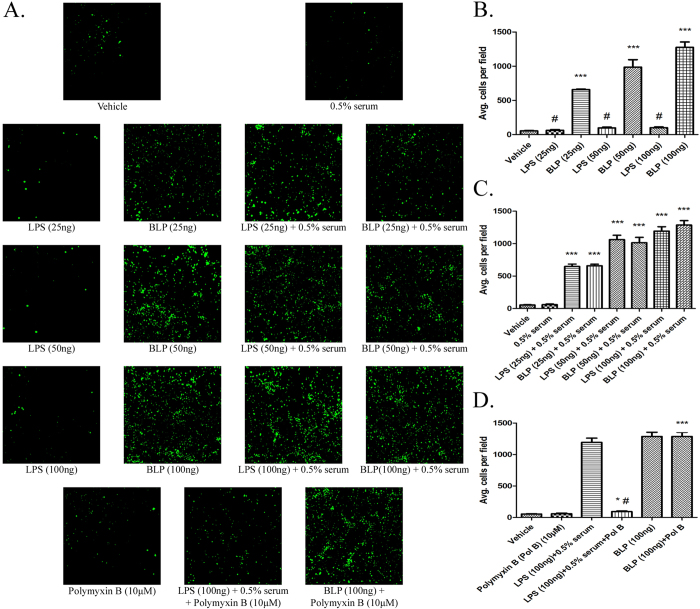
BLP primes human PMNs to adhere to gelatinized surface in a serum and polymyxin B – independent fashion. The PMNs loaded with calcein-AM in HBSS/A were treated respectively with vehicle, 0.5% serum, and increasing concentrations of LPS/BLP (25–100 ng/ml) in the presence/absence of 0.5% serum. The mixture was incubated for 60 minutes at 37 °C and the non-adherent PMNs were removed by washing with HBSS. The adherent PMNs were visualized under fluorescent microscope at a magnification of 40X (**A**). In a parallel experiment, to determine the effect of polymyxin B on PMN activation by LPS/BLP, PMNs were incubated either with Polymyxin B (10 μM) alone or along with LPS (100 ng/ml) + 0.5% serum or BLP (100 ng/ml) (**A**). The number of PMNs adhered in response to their activation by LPS/BLP in the absence (**B**) or presence (**C**) of 0.5% serum and the effect of polymyxin B on LPS/BLP induce PMN activation (**D**) were quantified by counting the cells per field using ImageJ software as explained under ‘Methods’, and the data shown are mean ± SEM (n = 3). *p ≤ 0.01; ***p ≤ 0.0001 when compared with the vehicle treated group and ^#^p ≤ 0.01 when compared to BLP treated group. These results represent the data obtained in three independent experiments.

Since PMNs are the first line of defense to be recruited at the site of infection^38^,^39^, we determined whether isolated human PMNs display proinflammatory responses to purified BLP *in vitro*. We treated isolated human PMNs previously loaded with fluorogenic calcein AM with increasing concentrations (25, 50 and 100 ng/ml) of LPS or BLP for 60 minutes at 37 °C in cell culture plates previously coated with 0.2% gelatin. Stimulated adhesion in this system reflects β2-integrin-dependent outside-in signaling^40^. The reaction mixture was also supplemented with 0.5% human serum as a source of CD14 since LPS requires it to elicit a response^40^. The fluorescent microscopic images (Fig. 2) show dose dependent adhesion of PMNs in response to activation by both BLP and LPS in the presence of serum when compared with the vehicle treated group. However, in the absence of serum, BLP showed significant adhesion while LPS showed a minimal response. Since polymyxin B blocks LPS-mediated activation and adhesion of PMNs^30^, we incubated PMNs with 10 μM polymyxin B for 30 minutes prior to incubating with BLP (100 ng/ml) or LPS (100 ng/ml) in the presence of 0.5% serum for 60 minutes at 37 °C. These results showed selective inhibition of LPS mediated activation and adhesion of human PMNs by polymyxin B, while BLP-mediated stimulation was insensitive to polymyxin B (Fig. 2). The two *E. coli* endotoxins are therefore independent PMN agonists.

### PMNs also synthesize PAF in response to BLP

**Figure 3:**
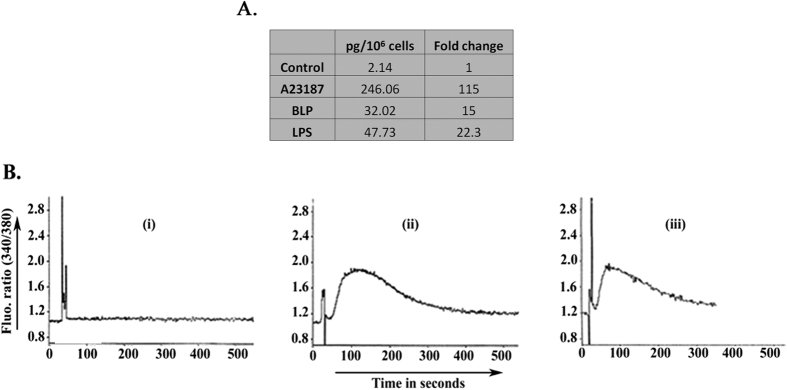
(**A**) PMNs synthesize PAF in response to distinct endotoxins. Isolated PMNs (10 × 10^6^ cells/ml) were treated with control, calcium ionophore A23187 (1 μM), LPS (5 μg/ml) + 0.5% serum or BLP (5 μg/ml) in cell culture plates previously coated with 0.2% gelatin and incubated at 37 °C for 60 minutes. A constant amount of [^2^H]PAF was introduced as internal standard and the lipids were extracted from the PMNs followed by aminopropyl column and HPLC purification as described under ‘Methods’. The fractions with PAF-like bioactivity were tested for the intracellular calcium accumulation in FURA-2 AM loaded PMNs (See below). The fractions with PAF-like activity were quantified by mass spectrometry as described in ‘Methods’. The results are expressed in terms of picograms of PAF synthesized per 10^6^ cells and represents the summation of 1-O-hexadecyl (C_16_), 1-O-octadecyl (C_18_), and 1-O-octadecenyl (C_18:1_) species. The result is a representative of two independent experiments. (**B**) Lipid extracts from PMNs stimulated by either LPS or BLP induced transient increases in intracellular calcium in naive PMNs. Isolated human PMNs were loaded with FURA-2 AM and washed twice with HBSS/A to remove unincorporated FURA-2 AM. 2.5 × 10^6^/ml cells were placed in a quartz cuvette and treated with (i) purified lipid fraction from control PMNs, (ii) Purified lipid fractions isolated from LPS treated PMNs (iii) Purified lipid fractions isolated from BLP treated PMNs. The tracings were recorded at dual excitation wavelengths of 340 and 380 nm and emission at 510 nm as a function of time. The increase in 340/380 ratio is associated with increased intracellular calcium. The result is a representative of two independent experiments.

PAF is a potent proinflammatory phospholipid mediator that exaggerates endotoxemia^41^,^42^,^43^. LPS induced synthesis of PAF by activated PMNs has been reported^40^. To determine if the same effect is induced by purified BLP, we treated the isolated PMNs with control, calcium ionophore A23187 (1 μM), LPS (5 μg/ml) + 0.5% human serum, or BLP (5 μg/ml). After incubation for 60 minutes at 37 °C, the purified polar phospholipids were quantified by mass spectrometry using [2H]PAF as internal standard with the results expressed as picograms of PAF synthesized per 10^6^ cells. This experiment (Fig. 3A) showed a 115 fold increase in PAF synthesis in response to calcium ionophore A23187, 22.3 fold increase in response to LPS and 15 fold increase in response to BLP. This result is in agreement with previously reported data^40^ using Pam3CysSK4 as synthetic TLR2 agonist. In a parallel experiment, purified lipid extracts derived from BLP and LPS treated cells exhibited increased intracellular Ca^2+^ concentrations in FURA-2 AM labeled PMNs while, equivalent lipid extract from control cells failed to show an increase in intracellular Ca^2+^ (Fig. 3B). This increase in intracellular Ca^2+^ concentrations was abolished by pretreating the cells with PAF receptor antagonists or by predigesting the lipid extract by recombinant PAF-acetylhydrolase (data not shown) as described earlier^44^. Both LPS and BLP stimulate PMNs to synthesize biologically active PAF to a similar extent.

### Purified BLP also stimulates expression levels of proinflammatory marker genes in the mouse macrophage cell line RAW264.7

**Figure 4:**
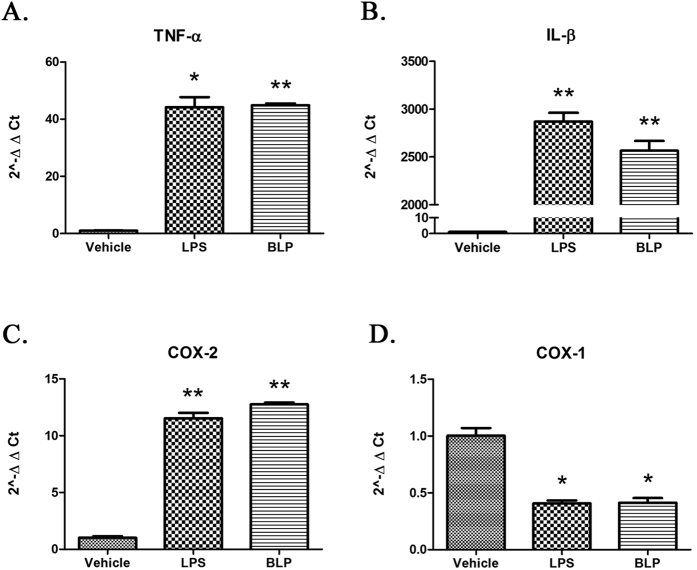
Induction of proinflammatory genes by BLP in RAW264.7 cells. RAW264.7 cells were cultured and maintained as described in ‘Methods’. 5 × 10^5^ cells/ml were treated for 2 hours with vehicle, LPS (100 ng/ml) or BLP (100 ng/ml). All the treatments were carried out in triplicates. After incubation, total RNA was extracted and real-time PCR analysis was carried out for (**A**) TNF-α, (**B**) IL-1β, (**C**) COX-2 and (**D**) COX-1. The results are represented in terms of 2^−∆∆CT^ with respect to GAPDH and the data shown are mean ± SEM (n = 3). *p ≤ 0.01; **p ≤ 0.001 when compared with the vehicle treated group. The results are representative of two independent experiments.

**Figure 5:**
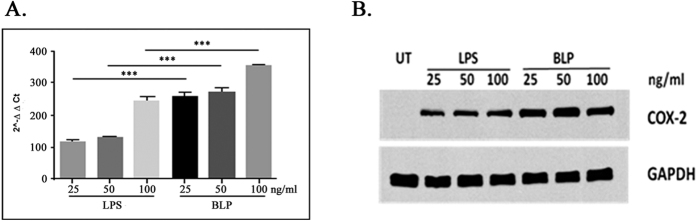
Concentration-dependent stimulation of COX-2 mRNA and protein expression in RAW264.7 cells. (**A**) Analysis for COX-2 mRNA by real-time PCR: RAW cells were stimulated with increasing concentrations (25 ng to100 ng/ml) of LPS or BLP for 6 hours. Total RNA from treated cells was isolated and real-time PCR was performed as described in ‘Methods’. Results are expressed as 2^−∆∆CT^, that is the expression of target gene relative to the housekeeping gene (GAPDH) and normalized to the untreated control. The data shown are mean ± SEM (n = 3), ***p ≤ 0.0001 when compared between LPS and BLP at same doses. (**B**) COX-2 protein expression in response to endotoxins: Lysates from the stated amounts of endotoxin-treated cells were prepared using RIPA buffer and immunoblots were developed using specific primary and appropriate secondary antibodies for COX-2 and GAPDH and visualized as described in ‘Methods’. The results represent data obtained in two independent experiments. One of the best among two full length blot is presented in Supplementary Figure S2.

LPS stimulates expression of proinflammatory marker genes such as TNF-α, IL-1β, and COX-2 in the mouse macrophage cell line RAW264.7^45^,^46^,^47^. Analyses of the expression of these genes show that BLP was as potent as LPS in stimulating the upregulation of each of these genes (Fig. 4A–C). The expression level of COX-1 gene was reduced when RAW264.7 cells were treated either with LPS (100 ng/ml) or BLP (100 ng/ml) when compared with the vehicle treated cells (Fig. 4D). Such a down-regulation of COX-1 gene is not an unusual phenomenon^48^ (See discussion). In experiments involving concentration-dependent effects of BLP on COX-2, we observed BLP to be more potent than LPS in stimulating both COX-2 mRNA and protein expression (Fig. 5). Since, LPS forms aggregates of varying sizes^49^, comparing the strength of agonists on molar basis would be difficult and hence, the agonist strengths are expressed as ng/ml.

### BLP toxicity is also augmented by D-GalN in Swiss albino mice

**Figure 6:**
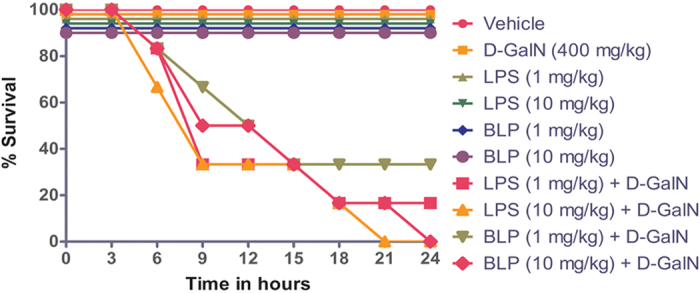
Effect of BLP administration in the presence/absence of D-GalN on the survival time of Swiss albino mice. 60 Swiss albino mice were divided into 10 groups containing six animals each and the mice were injected intraperitoneally with the stated concentrations of LPS or BLP in the presence/absence of D-GalN in a total volume of 500 μl. The survival time was monitored for 24 hours. The graph represents the percentage survival with respect to time and the result is a representative of three independent experiments. ***p ≤ 0.0001 when LPS/BLP + D-GalN injected groups compared to vehicle. (*Note – Survival in these groups was 100%. For representation purpose the survival % of these groups have been reduced to avoid overlapping and to distinguish clearly).

LPS toxicity is greatly increased by the presence of D-GalN in ways that are not currently understood^50^. We determined whether D-GalN specifically affects LPS signaling or generally augments inflammatory mechanisms by injecting groups of mice (n = 6) with vehicle, LPS, or BLP in the presence or absence of D-GalN (400 mg/kg body weight). The survival time was monitored for up to 24 hours. During this period the animals receiving LPS or BLP alone or in combination with D-GalN were anorectic after 60 minutes of injection. This was further accompanied by lethargy, somnolence and decreased general activity. The result shows, neither LPS nor BLP were lethal when injected alone at this concentrations of the toxin to this strain of mice (Fig. 6). Inclusion of D-GalN increased the toxicity of the lowest concentration (1 mg/kg) of both LPS and BLP we tested. The effect of D-GalN therefore is on the general response to endotoxin stimulation and not specific to LPS.

### Histological characterization of the proinflammatory properties of purified BLP

**Figure 7:**
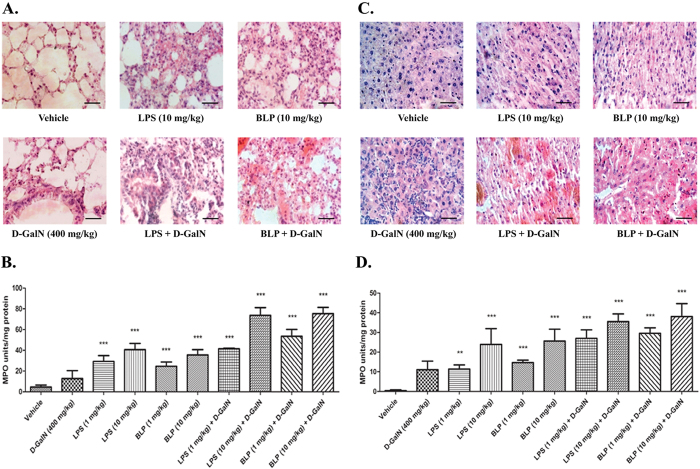
Effect of BLP administration on the histology and MPO levels of lungs and liver of Swiss albino mice. The lungs and livers were harvested from the animals injected with vehicle, LPS (10 mg/kg body weight) or BLP (10 mg/kg body weight) in the presence/absence of D-GalN (400 mg/kg body weight). The organs were processed and 5 μm sections were stained with hematoxylin and eosin before pictures were obtained at 200X magnification. Scale bar represents 200 μm. The PMN infiltration into the organs were indirectly estimated by quantifying the MPO activity, a PMN specific marker, in the tissue homogenates of (**B**) lung and (**D**) liver from respective groups as described in ‘Methods’. The results are presented as mean units/mg of protein ± SEM (n = 6). **p ≤ 0.05 and ***p ≤ 0.0001 when compared with the vehicle treated group. The results represent data obtained from three independent experiments.

Lungs and liver are the major organs affected during endotoxemia^51^,^52^. To test whether BLP similarly affects these organs, we harvested lungs and livers from animals immediately after the death or after 24 hours of injection. The animals injected with LPS or BLP alone survived and their lung sections (Fig. 7A) showed thickened alveolar septae, massive infiltration of cells of immune system, and accumulation of mucus when compared with the vehicle treated group. When LPS and BLP were injected in the presence of D-GalN, the lung sections showed vascular congestion and cytoplasmic vacuolization of the airways. The liver sections (Fig. 7C) of animals injected with LPS or BLP alone showed marked necrosis and highly distorted architecture in contrast to the normal lobular architecture of the vehicle treated group (Fig. 7C). Since D-GalN is hepatotoxic^53^,^54^ and sensitizes mice to LPS^55^,^56^, we determined whether this carbohydrate modulator also enhanced BLP-induced organ damage. We found (Fig. 7C) injecting animals with either LPS or BLP in the presence of D-GalN exaggerated the hepatic injury by generating areas of extensive hemorrhage. These results indicated that both LPS and BLP act in similar ways to severely insult lungs and liver.

**Figure 8:**
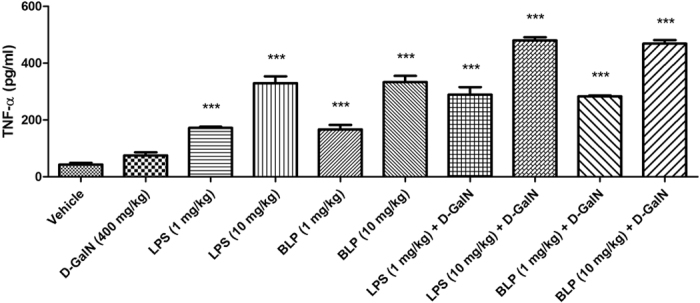
Effect of BLP administration on serum TNF-α levels in Swiss albino mice. Groups of Swiss albino mice (n = 6) were injected intraperitoneally with the stated concentrations of LPS or BLP in the presence/absence of D-GalN (400 mg/kg body weight) in a total volume of 500 μl. Blood was drawn from the animals by facial vein puncture 2 hours post injection and the serum TNF-α levels were quantified by sandwich ELISA. Values are presented as mean ± SEM (n = 6) expressed as pg/ml. ***P < 0.0001 when compared with the vehicle treated group. The result is a representative of data obtained from two independent experiments.

PMNs are first cells to be recruited to sites of injury and damage^38^,^39^. Therefore, quantifying MPO levels in the tissue homogenate^57^ signifies the extent of PMN infiltration^58^,^59^. We found a dose-dependent increase in MPO levels in the homogenates of lungs (Fig. 7B) and liver (Fig. 7D) of animals injected with LPS and BLP. MPO levels were even higher when LPS and BLP were injected in the presence of D-GalN. The MPO activity was higher in lung homogenates than within the corresponding liver homogenates. Little or no difference in the MPO activity was observed between LPS and BLP treated groups irrespective of the presence/absence of D-GalN. Generation of an inflammatory response, then, is not the way D-GalN promotes endotoxin damage to tissues. In contrast, the circulating cytokine TNF-α, implicated in animal models of endotoxemia^60^,^61^, was also increased by both LPS and BLP in a concentration-dependent fashion (Fig. 8). In addition, D-GalN enhanced TNF-α expression in response to each agonist, suggesting soluble cytokines, and not simply the level of inflammation, correlate to endotoxin-induced tissue damage.

### BLP – induced TNF-α production involves TLR2 activation

**Figure 9:**
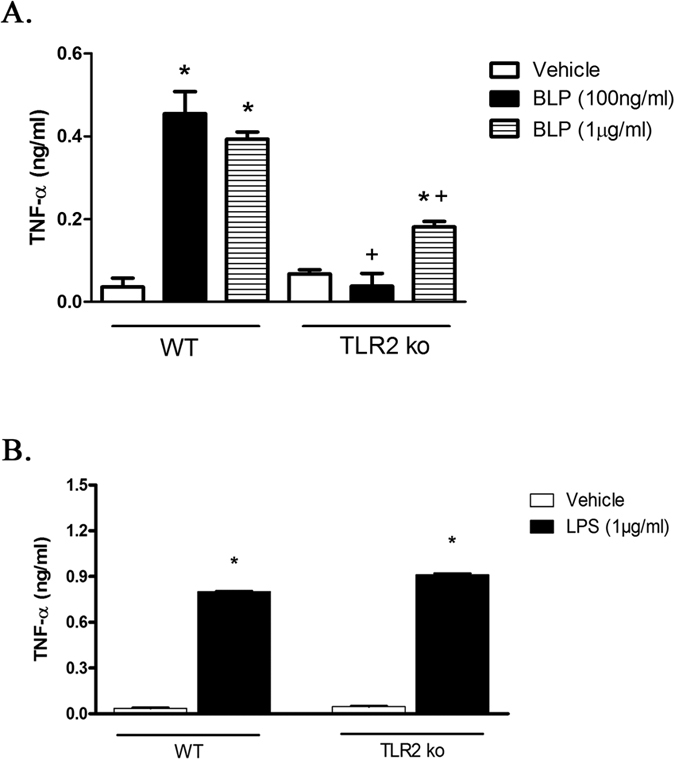
BLP induced TNF-α production in C57BL/6 mice peritoneal macrophages involves TLR2. Macrophages isolated from peritoneal cavity of wild type and TLR2^−/−^ mice (n = 3) were incubated with BLP (100 ng or 1 μg/ml) (**A**) or LPS (1 μg/ml) (**B**) for 24 hours. The supernatant was collected and assayed for TNF-α by ELISA. Each bar represents the mean ± SEM from 3 wells. *Denotes p < 0.05 compared to respective negative control while ^+^denotes p < 0.05 when compared to BLP. The data represents results obtained from two independent experiments.

To determine whether the proinflammatory signaling of BLP was mediated via TLR2, we treated macrophages derived from the peritoneal cavity of wild type (C57BL/6) and TLR2^−/−^ mice with BLP (100 ng/ml or 1 μg/ml) or LPS (1 μg/ml) and assayed for TNF-α. We found an increased production of TNF-α from the macrophages of wild type mice in response to BLP while, macrophages derived from TLR2^−/−^ mice produced minute amounts or no TNF-α (Fig. 9). However, the response of macrophages derived both from wild type and TLR2^−/−^ mice to LPS was intact to a similar extent. These results clearly demonstrate that BLP induced TNF-α production is through TLR2

## Discussion

Components of septic pathogenesis are induced by the widely employed bacterial endotoxin LPS, but bacterial LPS is not the sole endotoxin of Gram negative bacteria. Indeed, C3H/HeJ and C57BL/10ScCr mice that are hyporesponsive to LPS due to a mutation in their *tlr4* gene were just as sick as control mice when exposed to intact Gram negative bacteria^28^. This suggests the importance of other bacterial components besides LPS. Moreover, clinical trials involving anti-LPS approaches have not been successful in reducing the morbidity of sepsis^21^,^22^,^23^,^24^,^25^,^26^,^27^. Bacterial lipoproteins OspA and OspB isolated from *Borrelia burgdorferi* mimic the pathology of Lyme disease in mice^62^, suggesting bacterial lipoproteins do contribute to the response to whole bacteria. Similarly, genetic deletion of specific bacterial lipoproteins reduced virulence of intact *Yersinia pestis* in bubonic model of plague^63^,^64^,^65^,^66^,^67^,^68^,^69^. In this study, we used a *E. coli* mutant lacking BLP to show that the common lipoprotein of *E. coli* also contributes to the septic response of mice. The *in vivo* effects of BLP overlapped with LPS, but were not fully identical. In fact, in some responses BLP was even more potent than LPS (Fig. 5).

LPS and BLP are both exceedingly hydrophobic components of the *E. coli* outer membrane, are active in vanishingly small concentrations, and can contaminate each other’s preparation^29^. We establish that BLP is active *in vivo* as an endotoxin itself, both through the use of the BLP deficient *E. coli* strain, and by direct application of purified BLP demonstrably free of LPS contamination (<5EU/mg protein based on LAL assay). In addition, the BLP we purified and tested did not phenocopy the effects of LPS in whole animals or with inflammatory cells. In particular, we found BLP was considerably more effective in some assays than LPS (Fig. 5). We also found that BLP, unlike LPS, did not require serum, a source of CD14, and found that polymyxin B was fully effective in suppressing LPS- mediated effects, but left responses to BLP unaffected. Our results therefore show bacterial components other than LPS can contribute to endotoxemia.

We show BLP was equipotent with LPS in upregulating almost all the parameters that encompass septic inflammation such as induction of the key cytokines TNF-α and IL-1β in RAW264.7 cells (Fig. 4), β2 integrin mediated PMN adhesion (Fig. 2), and stimulation of the synthesis of inflammatory mediator PAF (Fig. 3A). In fact, BLP was even more potent than LPS in inducing COX-2 expression in RAW264.7 cells (Fig. 5). Both LPS and BLP down-regulated the expression of COX-1 gene to a similar extent. Such a down-regulation of COX-1 gene is not an unusual phenomenon, as it has been reported by Font-Nieves *et al*.^48^ where the activation of TLR4 by LPS led to the down-regulation of COX-1 gene in cultured asterocytes through MAPK independent but MyD88 dependent pathway^48^. Although, activation of TLR2 & TLR4 leads to similar mechanisms in terms of the involvement of MyD88^70^, whether down-regulation of COX-1 in response to TLR2 activation by BLP also involves MyD88 pathway needs to be examined.

Both LPS and BLP are endotoxins that evoke similar responses. For instance, the microarray analysis of HUVEC mRNA carried by Neilsen *et al*.^30^ showed that majority of the downstream events and outcomes are shared between BLP and LPS. However, BLP and LPS are not completely equivalent since, for example, IFN-γ-inducible protein-10, a CC-chemokine was specifically upregulated by LPS, but not by BLP. Similarly, Macrophage-activating lipopeptide-2 (MALP-2) from *Mycoplasma fermentas*, was capable of upregulating a battery of proinflammatory cytokines and chemokines in perfused mouse lungs via TLR2 of which, proinflmmatory marker tenascin C is uniquely upregulated by MALP-2, but not by LPS^71^. Thus, there are at least few proinflammatory markers that are uniquely expressed in response to TLR2 agonists.

BLP activates endothelial cells^30^, PMNs (Fig. 2) and macrophages *ex vivo* (Fig. 4)^33^, but the key element of the current study is that BLP itself has effects in Swiss albino mice that damage lung and liver, thereby promoting mortality. Among the tissues examined, lung tissue, expressing abundant TLR2^60^, was most severely affected by BLP. Moreover, TLR2^−/−^ macrophages did not respond to BLP while wild type macrophages produced TNF-α, suggesting the specificity of BLP acting through TLR2 (Fig. 9A). However, in these cells the response to LPS was found intact (Fig. 9B). We also noted a slight increase in the TNF-α production by TLR2^−/−^ macrophages incubated with the higher concentration BLP (1 μg/ml), suggesting receptors other than TLR2 may have weakly responded to BLP and this needs to be examined further. Nevertheless, the contribution of TLR2 for BLP-induced TNF-α production seems to be more important than others and BLP seems to have a higher affinity to TLR2. We also discovered that liver tissue is damaged by BLP and that D-GalN, that enhances liver damage by LPS, similarly enhanced both mortality and induction of the inflammatory cytokine TNF-α.

Thus, the pathway activated by bacterial lipoproteins playing an important role in the pathogenesis of sepsis has not been previously appreciated^72^. We conclude that besides LPS, lipoproteins such as BLP can also elicit inflammatory responses similar to LPS and this pathway should also be considered while attenuating the severity of septic challenge.

## Methods

### *Escherichia coli* strains

DH5α (BLP +ve) (MTCC#1652) was obtained from Microbial Type Collection Centre (MTCC), Chandigarh, India. JE5505 (BLP −ve) (CGSC#6672) strain was obtained from Coli Genetic Stock Center (CGSC), Yale University, New Haven, CT, USA. Although, *E. coli* JE5505 strain lacks BLP, its normal physiological properties of growth and multiplication are not impaired^73^.

### Animals

8–10 weeks old Swiss albino mice weighing 20–25 g were obtained from Central Animal Facility, University of Mysore after obtaining approval from Institutional Animal Ethics Committee (Approval No. UOM/IAEC/02/2012) of University of Mysore, India. All the experimental protocols involving animals were approved by from Institutional Animal Ethics Committee, University of Mysore, India. TLR2^−/−^ genetically deficient mice in a homogeneous C57BL/6 background and wild type mice (C57BL/6) weighing 20–25 g were provided by Federal University of Rio de Janeiro (UFRJ), Brazil. All the experiments were performed in accordance with Institutional ethical guidelines of animal welfare committee of the Oswaldo Cruz Foundation (CEUA/FIOCRUZ) for the care and use of laboratory animals, under license number LW 32/12. The animals were housed with adequate ventilation with free access to food and water.

### BLP lethality in Swiss albino mice

BLP +ve (DH5α) and BLP −ve (JE5505) strains of *E. coli* were cultured to stationary phase in LB broth on an orbital shaker at 37 °C for 12 hours. Although, the *E. coli* JE5505 strain lacks BLP, its growth and multiplication are unaffected^73^. The cells were harvested by centrifugation (5,000 × g, 10 minutes). The cell pellet was resuspended in PBS and the number of viable cells was determined by inoculating serially diluted bacterial suspensions on LB agar plates and counting the colony forming units (CFU) after overnight incubation at 37 °C. *In vivo* studies using either live or heat inactivated bacteria were performed according to the method followed by Sanders *et al*.^74^. Briefly, 1 × 10^9^ CFU of either bacterial strains were administered intraperitoneally to groups of mice (n = 6) before lethality was assessed after 72 hours. Alternatively, known amount of each bacterial strains were inactivated by incubating at 60 °C for 60 minutes and 1 × 10^9^ CFU of heat inactivated bacterial strains were injected intraperitoneally to groups of mice (n = 6) after confirming the efficient inactivation by overnight plating on LB agar plates. In some experiments, the bacteria were heat killed by autoclaving (120 °C and 15 lbs for 20 minutes).

### Purification of BLP from *E. coli* DH5α

Purification of BLP was carried out according to the method of Inouye *et al*.^75^ and Nielsen *et al*.^30^ with slight modifications. In brief, DH5α cells grown in LB broth for twelve hours at 37 °C were recovered by centrifugation and resuspended in S-buffer (10 mM sodium phosphate buffer pH 7.4 with 5 mM EDTA) containing 1 mM PMSF. The cells were lysed by sonication on ice (Vibra-Cell, Sonics & Materials Inc. Newtown, CT, USA) at a pulse rate of 9 second on and 5 seconds off mode for 30 minutes. Unbroken cells were removed by centrifugation at 3,000 × g for 10 minutes and the membranes were collected from the supernatant by centrifugation at 40,000 × g for 30 minutes at 4 °C (Sigma 3-30 KS, Osterode am Harz.Germany). The membrane pellet was resuspended in S-buffer and solubilized by adding SDS to a final concentration of 4% and β-mercaptoethanol to 0.5%. This mixture was boiled for 30 minutes and then stirred overnight at room temperature. The solubilized membrane fraction was supplemented with half the volume of 100 mM Tris-HCl buffer pH 8.5 along with another half the volume of 20 mM EDTA and pH of the mixture was adjusted to 8.0 with 1 M NaOH. To prevent the precipitation of SDS at low temperature, n-butanol was added to the above mixture. The contaminating proteins were removed from the above mixture by adding 4N sodium acetate buffer (pH 5.5) and adjusting the pH to 5.2 using acetic acid and was incubated on an ice bath for 20 minutes. The precipitate containing the contaminants was removed by centrifugation at 13,000 × g for 10 minutes at 4 °C. The supernatant from this step was supplemented with 1 M MgSO_4_ and 5% acetone, chilled on an ice bath for 20 minutes and then centrifuged at 13,000 × g for 10 minutes to remove the remaining contaminating insoluble proteins. The supernatant from this step was added with acetone to 30%, chilled on an ice bath for 20 minutes, and centrifuged at 13,000 × g for 10 minutes to pellet BLP. The BLP pellet was redissolved in S-buffer containing 1% SDS and fractionated 4 times with saturated phenol to remove the major contaminant LPS. Phenol phases from each step containing BLP were combined and supplemented with double the volume of acetone and diethyl ether to precipitate BLP. This mixture was incubated for 20 minutes at room temperature and the resulting BLP precipitate was sedimented by centrifugation at 13,000 × g for 10 minutes. The BLP pellet thus obtained was reconstituted using 10 mM sodium phosphate buffer (pH 7.4) containing 1% SDS. The 5% and 30% acetone fractionation steps were repeated twice to remove phenol. Trace amounts of phenol carried over during purification steps was further removed by molecular sieving on Sephadex G-50 (Sigma Chemicals Co., MO, USA) as described^76^ and the final precipitate was dissolved in 10 mM sodium phosphate buffer (pH 7.4) containing 1% SDS. The endotoxin content present in the purified BLP was assessed by Limulus amebocyte lysate (LAL) assay using Endochrome – K^TM^ kit (Charles River, SC, USA) as per manufacturer’s instructions.

### Biophysical characterization of purified BLP

MALDI-TOF-MS analysis was used to determine the molecular weight of the purified BLP. The spectrum for purified BLP was recorded on UltrafleXtreme MALDI TOF/TOF mass spectrometer (Bruker Daltonics, Bermen, Germany) using α-cyano-4-hydroxycinnamic acid as the matrix material and the spectrum was obtained using a nitrogen laser at 337 nm.

Circular dichroism (CD) was used to determine the secondary structure of purified BLP using Jasco J – 810 spectropolarimeter (MD, USA) fitted with neon lamp and calibrated with +D-10- camphor sulphonic acid ammonium salt. Dry nitrogen was purged before and during the measurements. CD measurements were carried in the far UV region in the range 190 nm to 260 nm using a 1 mm path length cell. The spectra of BLP were recorded at the speed of 50 nm/min. All scans were average of 3 runs. A mean residue weight of 115 was used for calculating molar ellipticity and secondary structural analysis employed the endogenous instrument program.

The fatty acid composition of purified BLP was determined by alkaline hydrolysis followed by gas chromatographic analysis of the liberated fatty acid. For this, the purified BLP was made lipid free as described by Inouye *et al*.^75^ and the resulting precipitate was dissolved in 10 mM sodium phosphate buffer (pH 7.4) containing 1% SDS. For fatty acid analysis, 1 mg of protein was subjected to alkaline hydrolysis by 0.5 M methanolic KOH for one hour at 65 °C. The liberated fatty acids were extracted into hexane and were then converted to fatty acid methyl esters according to the method of Morrison and Smith^44^. These methyl esters were injected into RTx-1 fused silica column (30 m × 0.32 mm ID) for GC analysis (Shimadzu, Model GC-2010, Kyoto, Japan) with eluting material detected by a FID detector. The optimum temperature conditions of injector (230 °C) and detector (250 °C) were maintained constant throughout the run, while the initial temperature of column oven was 120 °C and was increased by 5 °C per minute and finally held at 220 °C for 10 minutes. The total run time for each run was 30 minutes and the fatty acyl methyl ester peak was identified based on the standard fatty acid methyl esters.

### Isolation of polymorphonuclear cells (PMNs) and elucidating their responses to BLP

Blood was drawn after obtaining an informed consent from healthy human volunteers according to Institutional Human Ethics Committee (IHEC), University of Mysore, Mysore. The experiments were performed in accordance with the Institute’s ethical guidelines (Approval No. 107 Ph. D/2015-16). All the experimental protocols involving human participants were approved by the Institutional Human Ethics Committee, University of Mysore, which is authorized by the Indian Council of Medical Research (ICMR), New Delhi, India. The PMNs were isolated by dextran sedimentation and density gradient centrifugation over ficoll as described by Watanabe *et al*.^40^. The isolated PMNs (purity over 95%) were resuspended in HBSS containing 0.5% human serum albumin (HBSS/A).

For assessment of adhesion, the PMNs were loaded with calcein-AM (Invitrogen, OR, USA) to a final concentration of 1 μM prior to incubation for a period of 60 minutes at 37 °C with agonists in twelve well cell culture plates (Nest Biotechnology Co. Ltd., China) pre-coated with 0.2% gelatin. The PMNs were incubated with agonists in triplicate wells. Unbound PMNs were removed by washing twice with HBSS/A and the adherent PMNs were visualized and photographed at a magnification of 40x under fluorescent microscope (Nikon Eclipse TS 100, NY, USA) equipped with Q Imaging MicroPublisher 3.3 RTV camera. The number of cells adhered in each well was determined by counting the cells adhered in 10 randomly chosen fields using ImageJ software and then calculating the average number of cells adhered per filed. Results are expressed in terms of mean ± SEM.

The measurement of intracellular Ca^2+^ in PMNs was carried out as described previously^77^. Briefly, the isolated PMNs were labeled with FURA-2 AM (Invitrogen, OR, USA) at a final concentration of 1 μM, incubated in dark at 37 °C for 45 minutes and washed with HBSS/A to remove unincorporated FURA-2 AM. The washed cells were resuspended in HBSS/A and for each assay 2.5 × 10^6^ cells/ml were placed in a quartz cuvette in the presence of vehicle/agonist. The calcium tracings were recorded in Shimadzu spectrofluorophotometer with dual excitation wavelengths of 340 nm and 380 nm and emission at 510 nm as a function of time.

The platelet-activating Factor (PAF) synthesized by the PMNs was quantified as described by Watanabe *et al*.^40^. In brief, 10x10^6^ cells were stimulated with stated agonists on gelatin coated cell culture plates for one hour. A constant amount of [^2^H]PAF was introduced as internal standard. Lipids were extracted in methanol and transferred to teflon-capped glass tubes and mixed with appropriate amounts of chloroform according to the method of Bligh and Dyer^78^. Phospholipids from total lipids were separated using aminopropyl columns (J.T. Baker Inc, NJ, USA) as described previously^77^,^78^,^79^. The polar phospholipids were further fractionated by reversed-phase high-performance liquid chromatography (RP-HPLC) (ODS silica, 250X4.6 mm, Microsorb MV, Rainin Instruments Co., MA, USA) as described earlier^77^. PAF-like activity in the fractions eluting from the reversed-phase column was defined after drying and reconstituting an aliquot in HBSS/A and analyzing their effect on FURA-2 AM labeled PMNs as described above. Fractions with PAF-like activity were pooled, solvent was evaporated under N_2_ gas, resuspended in HBSS/A, and diacyl lipids were removed by treating with 50 units of phospholipase A_1_ from *R. arrhizus* (Sigma Chemicals Co, Mo, USA) in HBSS/A overnight at 37 °C. PAF-like ether phospholipids were recovered from the above mixture by passing through aminopropyl columns and solubilized in methanol for analysis by electrospray ionization/MS/MS.

### Proinflammatory mRNA in RAW264.7 cells

RAW264.7 macrophage cells were obtained from ATCC and maintained as described previously^80^. RAW264.7 cells (5 × 10^5^ cells/ml) cultured in growth media were treated for 2 hours with vehicle, LPS (100 ng/ml) (Sigma Chemicals Co., MO, USA) or BLP (100 ng/ml). In studies involving concentration-dependent effects of LPS and BLP on COX-2 mRNA expression, the RAW264.7 cells were stimulated with increasing amounts of BLP and LPS (25 ng to 100 ng/ml) for 6 hours. Following incubation, total RNA from treated cells was isolated using Isol-RNA lysis reagent (Prime Inc., MD, USA) according to manufacturer’s protocol. Reverse transcription-PCR was performed using 1 μg of total RNA with random primers using the High Capacity cDNA Reverse Transcription Kit (ABI-Life technologies, CA, USA) in a PTC-100 thermal cycler as per manufacturer’s protocol. Real time PCR was performed using a panel of proinflammatory gene markers; TNF-α, IL-1β, COX-1 and COX-2. Results were expressed as 2^−∆∆CT^, that is the expression of target gene relative to the house-keeping gene GAPDH and normalized to the vehicle control.

### COX-2 protein expression analysis

To study COX-2 protein expression, RAW264.7 cells were treated for 6 hours with LPS or BLP (25 ng to 100 ng/ml). Lysates were prepared using Radio-Immunoprecipitation Assay (RIPA) buffer and immunoblots were developed using specific primary and appropriate secondary antibodies for COX-2 (Cayman Chemicals, MI, USA) and GAPDH (Fitz Gerald Industries, MA, USA). The bands were visualized using SuperSignal West Pico ECL assay kits.

### *In vivo* studies using BLP in Swiss albino mice

The stated concentrations of BLP and LPS were administered intraperitoneally in the presence/absence of D-GalN (400 mg/kg) to Swiss albino mice. The survival rate was monitored for a period of 24 hours. Clinical signs manifested during this time period were visually monitored and all the animals were euthanized after 24 hours of injection. Lungs and liver from the animals were collected for histological analysis as described below.

### Histological Analysis

Resected lungs and livers were fixed in Bouine’s fixative solution (picric acid, formaldehyde, glacial acetic acid, 30:10:2) for 24 hours and then the tissues were dehydrated with increasing concentrations of ethanol and embedded in paraffin wax. 5 μm thick sections were obtained by microtome (R. Jung AG, Germany) and stained by classical hemotoxylin and eosin differential staining protocol.

### Quantification of tissue myeloperoxidase (MPO)

The amount of PMN infiltration into the organs was indirectly estimated by quantifying the tissue MPO levels that was carried out according to the method of Bradley *et al*.^81^. Briefly, the tissues were homogenized in 50 mM sodium phosphate buffer (pH 6.0) containing 0.5% hexadecyltrimethyl ammonium bromide (HTAB) and the homogenates were subjected to sonication and repeated freeze-thawing. The homogenates were centrifuged at 40,000 × g for 15 minutes at 4 °C. Aliquots (100 μl) of these supernatants were mixed with 2.9 ml of 50 mM sodium phosphate buffer (pH 6.0) containing 167 μg/ml of o-dianisidine (SRL, Mumbai, India) and 0.0005% H_2_O_2_. The change in absorbance at 460 nm was recorded in a UV-Visible spectrophotometer (Biomate 3S, Thermo Scientific, USA) and the MPO activity was expressed in units, where one unit of MPO activity is defined as one micromole of peroxide degraded per milligram of protein per minute at 25 °C.

### Measurement of serum TNF-α levels in Swiss albino mice

The concentration of circulating TNF-α was measured in serum from blood collected 2 hours post injection. This blood was allowed to clot at room temperature, centrifuged at 1000 × g for 15 minutes and TNF-α levels were quantified using ELISA kit (USCN Life Sciences Inc., Buckingham, UK) according to the manufacturer’s instructions.

### Measurement of TNF-α in peritoneal macrophages derived from TLR2^−/−^ mice

In order to firmly establish the action of BLP is exclusively via TLR2, we used macrophages from TLR2^−/−^ as well as wild type mice. However, to accomplish this, we had to use C57BL/6 mice strain since TLR2^−/−^ are not available in Swiss albino background. Macrophages from the peritoneal cavity of wild type (C57BL/6) or TLR2^−/−^ mice (n = 3) were harvested with sterile RPMI-1640 medium and were transferred to separate wells. Macrophages were allowed to adhere to culture plates for 2 hours at 37 °C in a 5% CO_2_ atmosphere, and washed twice vigorously with PBS to remove non-adherent cells. Macrophages (1 × 10^6^ cells/ml) were adhered in cover slides within culture plates (24 wells) overnight with RPMI-1640 medium containing 2% fetal bovine serum (FBS). Macrophages were treated either with BLP (100 ng or 1 μg/ml) or LPS (1 μg/ml) for 24 hours at 37 °C in 5% CO_2_ atmosphere. The cell free supernatants were recovered and quantified for TNF-α using ELISA kit (BD Biosciences, NJ, USA) as per manufacturer’s instructions.

### Statistical analysis

The animal experiments are representatives of 2 or more independent experiments and the statistical significance among groups was determined by log-rank test. Analysis for inflammatory markers in RAW264.7 cells were performed in triplicates and the numerical data are expressed as mean ± SEM. An un-paired two-tailed t-test was used to compare the mean for each treatment group with the mean of the control group. All other results were analyzed using one-way analysis of variance (ANOVA) wherever applicable.

## Additional Information

**How to cite this article**: Lakshmikanth, C. L. *et al*. *Escherichia coli* Braun Lipoprotein (BLP) exhibits endotoxemia – like pathology in Swiss albino mice. *Sci. Rep.* **6**, 34666; doi: 10.1038/srep34666 (2016).

## Supplementary Material

Supplementary Information
