# Supplementary material for: Escherichia coli Braun Lipoprotein (BLP) exhibits endotoxemia – like pathology in Swiss albino mice
Source: Sci Rep. 2016 Oct 4;6:34666. doi: 10.1038/srep34666 (PMC5048175; doi:10.1038/srep34666)

***Escherichia coli* Braun Lipoprotein (BLP) exhibits endotoxemia – like pathology in Swiss albino mice**

Chikkamenahalli Lakshminarayana Lakshmikanth^1^, Shancy Petsel Jacob^1^, Avinash Kundadka Kudva^2^, Calivarathan Latchoumycandane^3^, Puttaraju Srikanta Murthy Yashaswini^4^, Mosale Seetharam Sumanth^1^, Cassiano F. Goncalves-de-Albuquerque^5^, Adriana R. Silva^5^, Sridevi Annapurna Singh^4^, Hugo C. Castro-Faria-Neto^5^, Sandeep Kumble Prabhu^2^, Thomas M. McIntyre^3^ and Gopal Kedihithlu Marathe^1*^

^1^ Department of Studies in Biochemistry, University of Mysore, Manasagangothri, Mysore – 570 006, Karnataka, India.

^2^ Department of Veterinary and Biomedical Sciences, Center for Molecular Immunology and Infectious Disease and Center for Molecular Toxicology and Carcinogenesis, 115 Henning Building, The Pennsylvania State University, University Park, PA 16802, USA.

^3^ Department of Cellular and Molecular Medicine, Cleveland Clinic Lerner Research Institute, 9500 Euclid Avenue, Cleveland, Ohio 44195, USA.

^4^ Department of Protein Chemistry & Technology, Central Food Technological Research Institute/CSIR, Mysore – 570 020, Karnataka, India.

^5^ Laboratótio de Imunofarmacologia, Instituto Oswaldo Cruz, Fundação Oswaldo Cruz, Rio de Janeiro, RJ, 21045-900, Brazil.

^*^ To whom correspondence may be addressed: Gopal Marathe K, Department of Studies in Biochemistry, University of Mysore, Manasagangothri, Mysore, Karnataka, India. Tel.: (91) 9686423624; E-mail: [marathe1962@gmail.com](mailto:marathe1962@gmail.com)

**SUPPLEMENTARY INFORMATION**

**Purification and biophysical characterization of BLP from *E.coli* DH5α.** *E.coli* DH5α cells grown in LB broth yielded 3g cells per liter of culture media. Typically, the total yield of purified BLP from 200g cells was ~20mg, quantified according to the method of Lowry et.al., ^S1^ and also by BCA method. Each step of purification was monitored by Tris-Tricine-SDS-PAGE according to the method of Schägger et. al. ^S2^ on a 10% gel, with the final purified fraction presenting as a single band after silver staining (Supplementary Figure S1A). In each BLP preparation, the LPS content as determined by LAL assay was found to be less than 5EU/mg of protein, a level not known to interfere in any one of the lipoprotein mediated assays. The purified lipoprotein was subjected to biophysical characterization to confirm that the lipoprotein was indeed BLP. The MALDI-TOF-MS data confirmed the molecular weight of the purified protein to be 7.14kDa (Supplementary Figure S1B). This is in agreement with the mass spectral data published by Pittenauer et.al., ^S3^. The CD spectra obtained for purified BLP (Supplementary Figure S1C) showed the presence of 82% α-helix and the remaining 18% as random coils – a value comparable to previously published report ^S4^. Since, BLP contains palmitate, we subjected the purified BLP for fatty acid analysis by GC after delipidation and converting the fatty acids to respective methyl esters. The GC spectra obtained (Supplementary Figure S1D) showed a single peak at the retention time of 8.315 minutes corresponding to the time obtained for standard palmitic acid methyl ester under same experimental conditions.

**Supplementary Figure Legend**

**Supplementary Figure S1: Purification and biophysical characterization of BLP.** BLP was purified from *E.coli* DH5α cells as described in ‘Methods’. Each step of purification was monitored by Tris-Tricine-SDS-PAGE electrophoresis on a 10% gel that was stained with silver nitrate (A). [Lane 1, crude lysate; 2, solubilized membrane; 3, supernatant after 4N sodium acetate treatment; 4, supernatant after 5% acetone treatment; 5, redissolved pellet after 30% acetone treatment; 6, supernatant after 30% acetone precipitation; 7 & 8, BLP after each round of phenol treatment and precipitation by 30% acetone treatment; 9, BLP after molecular sieving using Sephadex-G-50 (10µg); 10, Molecular weight marker]. (B) The mass spectral analysis of purified BLP by MALDI-TOF-MS. The *inset* shows the endotoxin content in purified BLP based on LAL assay (C) Secondary structure determination of BLP by Circular Dichroism. (D) Fatty acid composition of purified BLP by Gas Chromatography. All the results are representative of 3 independent experiments.

**Supplementary Figure S2:** Full length image of western blot from figure 5. Region of interest is highlighted and presented as cropped image in figure 5. COX-2 protein expression in response to endotoxins: Lysates from the stated amounts of endotoxin-treated cells were prepared and immunoblots were developed using specific primary and appropriate secondary antibodies for COX-2 and GAPDH and visualized as described in ‘Methods’

**Supplementary References:**

S1 Lowry, O. H., Rosebrough, N. J., Farr, A. L. & Randall, R. J. Protein measurement with the Folin phenol reagent. *J Biol Chem* **193**, 265-275, (1951).

S2 Schagger, H. Tricine-SDS-PAGE. *Nat Protoc* **1**, 16-22, (2006).

S3 Pittenauer, E. *et al.* Characterization of braun's lipoprotein and determination of its attachment sites to peptidoglycan by (252)Cf-PD and MALDI time-of-flight mass spectrometry. *J Am Soc Mass Spectrom* **6**, 892-905, (1995).

S4 Inoyye, S. *et al.* Lipoprotein from the outer membrane of Escherichia coli: purification, paracrystallization, and some properties of its free form. *J Bacteriol* **127**, 555-563, (1976).

**Supplementary Figure S1**


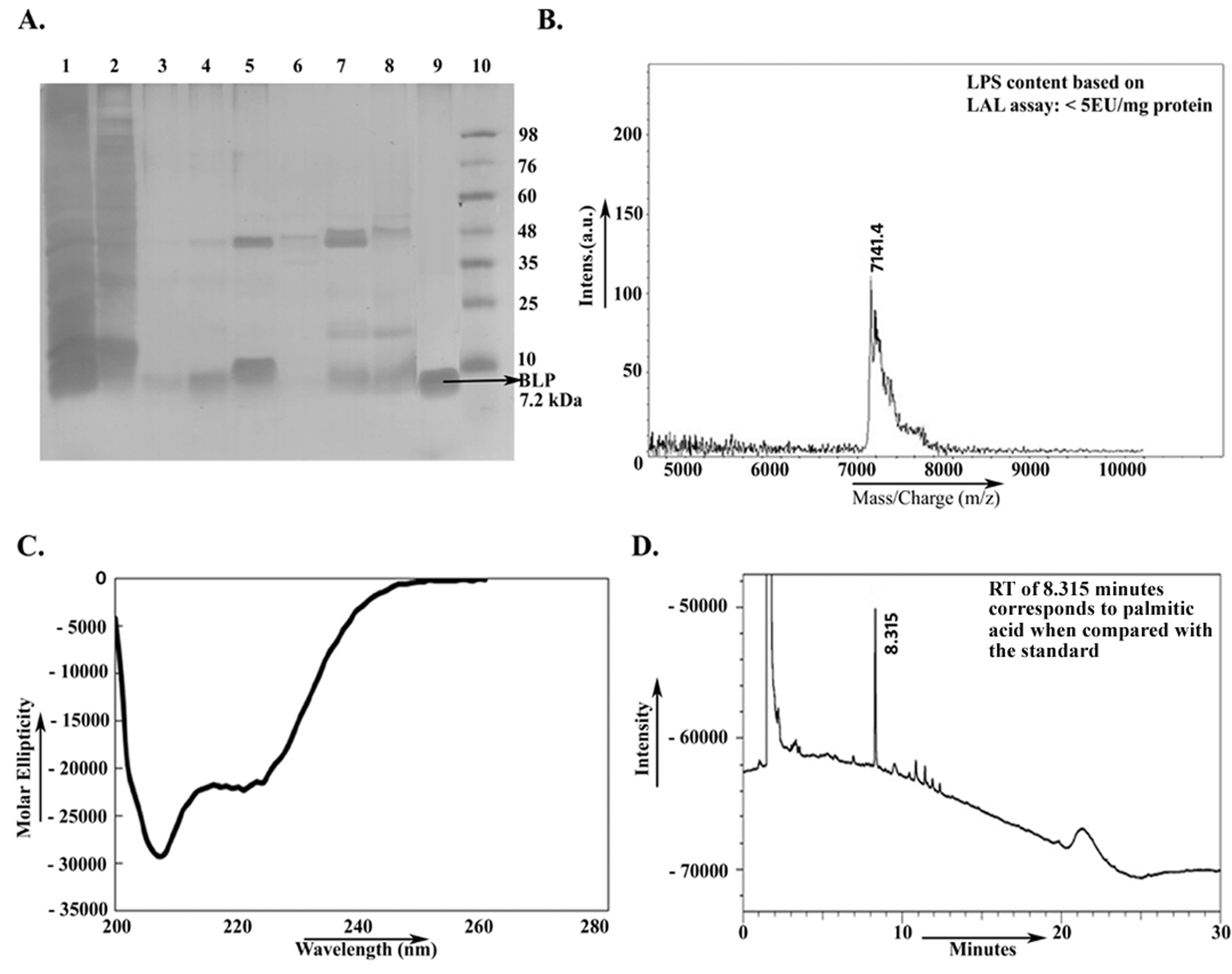


**Supplementary Figure S2**


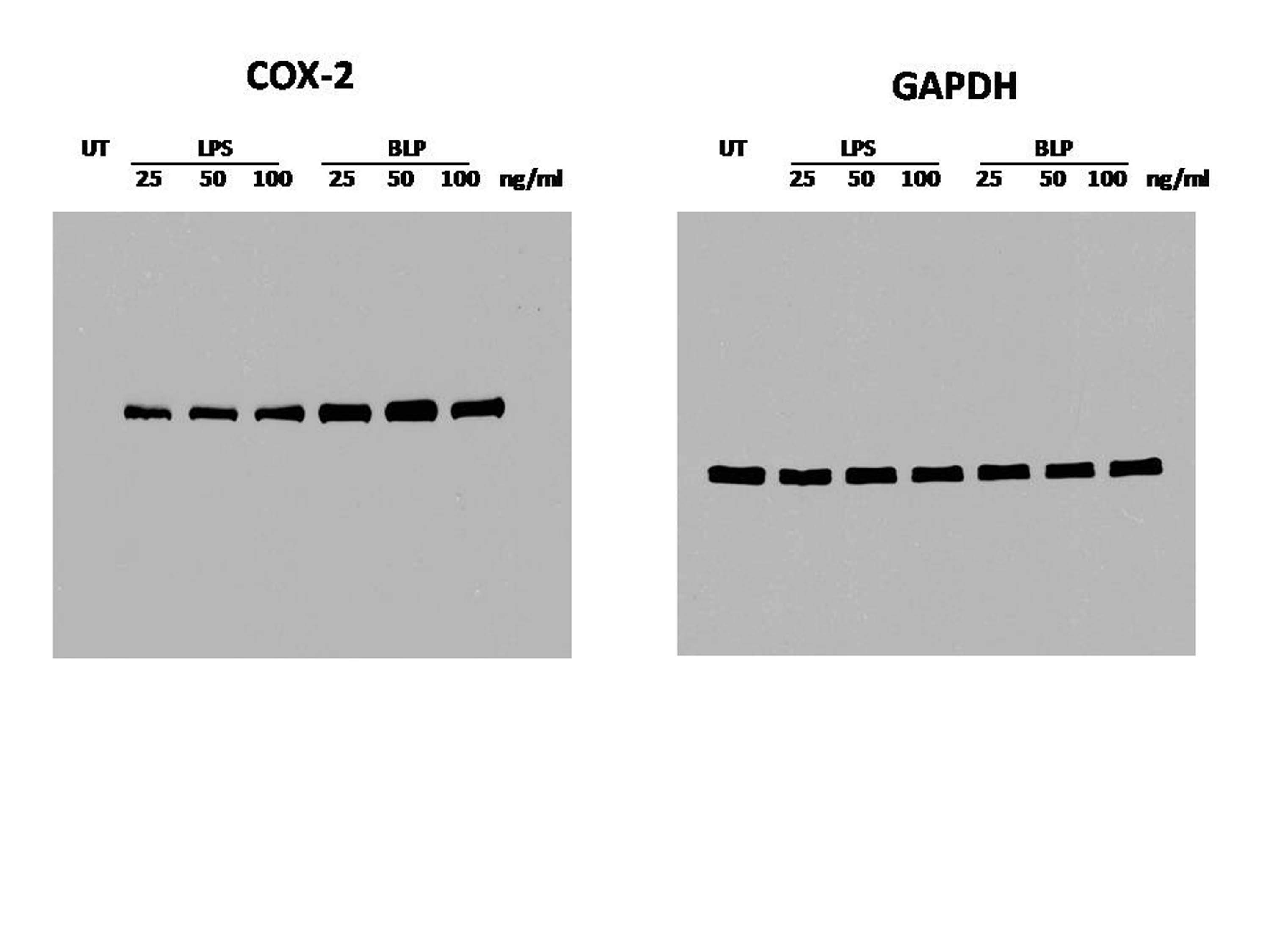

Supplement: Supplementary Information [file srep34666-s1.doc]
